# Performance Evaluation of AlphaSensor Radon Modules Under Real-World Conditions

**DOI:** 10.3390/s26051432

**Published:** 2026-02-25

**Authors:** Atanas Terziyski, Ludmil Tsankov, Stoyan Tenev

**Affiliations:** 1 Faculty of Chemistry, Plovdiv University, 4000 Plovdiv, Bulgaria; 2 Faculty of Physics, Sofia University, 1164 Sofia, Bulgaria

**Keywords:** radon monitoring, AlphaSensor, AlphaGUARD, performance evaluation, comparative study, uncertainty analyses

## Abstract

This study compares a set of 43 AlphaSensor units produced by RadonTec GmbH, Wittislingen, Germany against the AlphaGUARD 1000PF (Bertin Technologies, Montigny-le-Bretonneux, France), which is used as a reference monitor. During this study, around 16 k integrated measurements were conducted. The concentration range varied between 10 and 20 k Bq/m^3^. Multiple key performance indicators, such as sensitivity, uncertainty, background, linearity, and temporal response, were evaluated using a variety of statistical approaches. The results confirm the manufacturer’s claim of 10% or lower uncertainty in comparison with AlphaGUARD. We tentatively suggest individual calibration factors and methodologies. Our conclusion is that the AlphaSensor and commercial devices based on it, such as AlphaTracer, are affordable and applicable for home use. With modest additional hardware, AlphaSensors are also a good option for scientific studies involving the deployment of large monitoring networks.

## 1. Introduction

The methodology for assessing airborne radon has transitioned from static, cumulative measurements toward high-resolution dynamic monitoring. This development is motivated by limitations of traditional passive detectors, which provide only a retrospective, time-averaged value of radon-222 (^222^Rn) exposure [1]. Such integrated methods are often insufficient for characterizing health risk because they fail to resolve the sharp temporal fluctuations and high-frequency variations driven by environmental factors [2]. In contrast, electronic radon monitors facilitate a more detailed assessment by recording data at precise, regular intervals [1,3]. This temporal resolution is particularly critical for monitoring radon in soil gas, where transport mechanisms are highly sensitive to meteorological drivers like atmospheric pressure and ambient temperature [4,5].

The requirement for quantitative precision spans both public health management and geophysical research [6]. Accurate observations in terms of national action levels are essential for making informed mitigation decisions, as even minor measurement uncertainties can lead to a misinterpretation of safety guidelines [1]. In geophysics, radon serves as a significant tracer for seismic activity. Crustal strain causes micro-fractures that increase radon release from soil into the atmosphere [4]. However, detecting these seismotectonic signals is challenging because they are frequently obscured by atmospheric “noise” [7]. To successfully isolate tectonic anomalies from meteorological interference, researchers must deploy a network of reliably comparable instruments that maintain metrological consistency across all nodes [8].

The technical selection of sensors is fundamental to ensuring field reliability. While pulsed ionization chambers offer high sensitivity, they are notably vulnerable to environmental instabilities, such as high humidity and physical vibrations, which can result in signal underestimation or the generation of spurious pulses [7]. On the other hand, Lucas cell scintillation technology provides a more robust alternative for field monitoring. These sensors are characterized by their resilience to fluctuations in humidity and temperature, and they maintain a stable counting capability across an extensive measurement range, occasionally reaching 1 MBq/m^3^ [4]. These attributes make Lucas cells, such as those used in the RadonTec GmbH AlphaSensor, particularly suitable for the demanding conditions of subsurface and indoor monitoring [9].

The characterization of measurement instruments can be approached through two general methodological frameworks. Often, active experiments are carried out that presume creating and maintaining strictly controlled laboratory conditions to evaluate the system. This method allows researchers to keep studied characteristics within a desired range, though it requires the use of sophisticated and expensive equipment [1,2,3,7]. Another approach involves performing passive observation that includes monitoring the investigated system under real environmental conditions using in parallel high-precision reference instruments. While simpler to implement, this method carries the inconvenience that data may fall outside the intended calibration range [4,5].

In our assessment, the observational approach better reflects a sensor’s functional performance in practical, real-world applications. The present study, which conducts a comparative analysis of 43 AlphaSensors, is based on such observations to ensure the reliable operation of the sensing grid in situ.

Building a robust monitoring infrastructure requires a rigorous process of referencing and refinement. Independent validation is necessary to verify manufacturer specifications and protect against potential performance deviations [1]. Our methodology for the comparison of 43 AlphaSensors involves benchmarking the entire set against a high-grade AlphaGUARD device, which serves as a global benchmark for performance [9]. By deriving individual correction factors for each unit to eliminate inter-device bias, we ensure that the sensors remain reliably comparable [8]. This metrological validation satisfies the quality assurance requirements necessary for both professional residential radioprotection and the identification of geodynamic signals associated with seismic processes [1,6].

## 2. Materials and Methods

### 2.1. Experimental Setup

**Figure 1:**
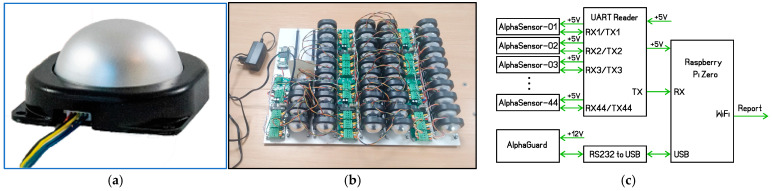
(**a**) Photo of AlphaSensor module; (**b**) panel with all tested AlphaSensors and electronics; (**c**) schematic diagram of the setup.

Forty-three AlphaSensor modules (hereafter denoted ASs) by RadonTec, Wittislingen, Germany [10], were examined in the present study. Each module (Figure 1a) consists of a Lucas cell-type alpha-scintillation detector and front-end electronics which counts pulses from the detector, calculates the radon activity concentration as a 60 min moving average from the last six ten-minute measurements and transmits the data on request by serial interface. The digital response of the sensor is internally refreshed every 10 min.

An AlphaGUARD 1000PF portable device (or simply AG) by Bertin Technologies, Montigny-le-Bretonneux, France [11], was used as a reference monitor for the radon concentration activity. It is based on a pulse ionization chamber with 0.56 L active volume and operates in flow-through mode by means of a special pump (AlphaPUMP) with 1 L/min debit. AlphaGUARD is also provided with additional sensors for some basic environmental parameters (ambient temperature, atmospheric pressure and relative humidity) and a real-time clock (RTC). It measures radon concentration continuously and reports data every 10 min on request via serial interface. All AlphaGUARD family instruments are carefully calibrated by the manufacturer with reference to national radon standards.

Some basic technical parameters of the sensors used for radon monitoring are summarized in Table 1, as provided by the manufacturers.

To collect data from all sensors within a short time interval, a dedicated data acquisition system with three-level architecture was developed (Figure 1c):

1. First-level processor (UART Reader): All AlphaSensors are read at 10 min intervals;
2. Master processor (Raspberry Pi Zero, Raspberry Pi Foundation): It receives the data from the first-level processor and those from AlphaGUARD, aggregates them and transmits them via Wi-Fi to a remote server;
3. Remote server (https://meter.ac): Synchronized with UTC time; it sends a request command to the master processor every 10 min, collects all data and supports archive and online visualization in real time.

All studied AlphaSensors and associated electronics were mounted on a 35 × 45 cm plastic panel (Figure 1b).

### 2.2. Test Site

An outbuilding in Sarnegor village, Bulgaria (N 42.458, E 24.937, 408 m a.s.l.), was used as the test site. It is suitable for radon monitoring due to the increased radon exhalation rate from the soil which has been identified in previous studies. The room has brick walls, concrete floor and no heating/ventilation facilities, so its environmental conditions are close to the outside ones.

The experimental setup was situated in the middle of the room (with a volume of about 15 m^3^) on a table 0.8 m above the floor. AlphaGUARD operates in a flow-through open loop mode. The air flow is blown by AlphaPUMP (Bertin Technologies, Montigny-le-Bretonneux, France) and enters into the device through a glass fiber filter provided by the producer which allows radon gas but stops radon progeny. The inlet pipe of the pump was placed near the center of the panel with AlphaSensors to achieve same radon exposure for all monitors. The entrance door of the room was kept firmly closed during the experiment. The room is, however, not hermetically isolated from the environment, so the weak air exchange between in- and outside air prevents the radon stratification on the bottom. The radon concentration inside the building changes substantially and is conditioned by the temperature and humidity gradients. The nearby outdoor environment was also monitored by an automatic meteorological station.

### 2.3. Data Acquisition Procedure

The main radon exposure cycle took place between 27 February and 19 April 2025. More than 10,000 samples of 600 s each were collected and recorded for each sensor. No data loss has been observed due to communication problems.

The weather during this period was highly variable: temperature ranged from 3 to 24 °C, atmospheric pressure was between 947 and 991 hPa, and the relative humidity was between 56 and 74 percent. As a result, the radon activity concentration in the test room air changed in wide intervals, sometimes jumping rapidly up to 20 kBq/m^3^.

After the end of the first cycle, the experimental equipment was removed from the test room, ventilated, and installed in an airtight 200 L steel vessel. A small sealed flange served for passing the power cables and the wireless antenna. The aim of this setup was to prevent radon ingress from outside in order to estimate the intrinsic background of AlphaSensors and that of AlphaGUARD (which is due to the radiation arising from the long-lived radionuclides). This cycle continued from 20 April 2025 to 12 May 2025. The decay curve of the initially available radon in the vessel was monitored, recorded and used for the background evaluation. Data collected within that period were added to those of the previous cycle and used in the general analysis.

Another radon exposure cycle was conducted between 17 September and 6 October 2025, because the data acquired so far were very scarce in the activity interval 100–800 Bq/m^3^. Radon monitoring was restarted in the test room and the ventilation was intentionally regulated (by half-opening the door and watching the effects on radon concentration) so that most of the data fell within the desired range.

Altogether, about 16,000 parallel measurements of 10 min each of the radon activity concentration with AlphaSensors and AlphaGUARD were obtained during the experiment.

**Figure 2:**
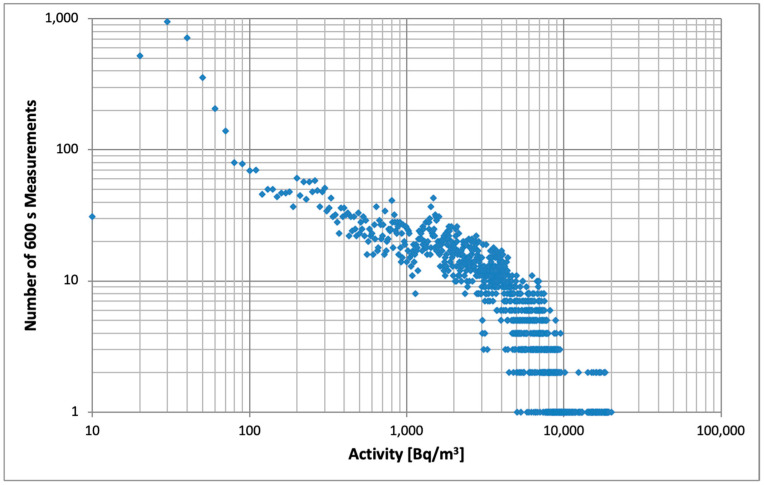
Activity distribution of measurements. The dots represent the number of 600 s measurements within the range obtained from each detector during the experiment.

A histogram of activity distribution of the measurements is displayed in Figure 2. It is seen that the activity interval between 10 and 4000 Bq/m^3^ includes sufficient events across the range for detailed statistical analysis.

### 2.4. Data Analysis

#### 2.4.1. Measurement Uncertainty

The main sources of statistical uncertainty include counting statistics of radioactive decay, measurement uncertainty due to perturbations from the environment, and some model uncertainty (inaccurate relationship between the registered signal strength and the radon concentration). The producers of AlphaGUARD use proprietary algorithms to estimate radon concentration and its overall uncertainty, and both values are readily reported by the firmware. The AS module, however, does not offer uncertainty calculation. It was estimated using the sample standard deviation of radon concentration values reported at the same time from all tested AlphaSensors. Its behavior as a function of activity is very similar to that of AG’s uncertainty and both can be well characterized by a simple power function.

In all subsequent operations on the data, the corresponding estimated uncertainties are transformed according to the uncertainty propagation law.

#### 2.4.2. Auxiliary Steps

The AlphaSensor data are reported to the user as a moving average over the last six 10 min measurements. This procedure somewhat reduces the effects of the low sensitivity of AS but blurs the radon concentration dynamics. In order to correctly compare the AS results with those of AG, however, a 60 min averaging procedure was also applied to all AG data (radon concentration and environmental parameters). The AG concentration uncertainty was accordingly transformed.

In the following sections, some results are represented for better visibility by aggregation of data (either in time or in the activity domain) or by decimation (e.g., one point per hour). In all cases, the results are obtained by processing the whole data set, and uncertainties shown in the figures are respectively recalculated.

#### 2.4.3. Time Synchronization

All recorded data must be reduced to a common time scale. The AlphaGUARD device has built-in RTC, and upon request reports two timestamps: the current time and the end of the last measurement cycle. This allows its synchronization with UTC. The AlphaSensor module, however, does not include an RTC and the end of its measurement cycle is unknown to the end-user. Although data from all sensors joined in a record are obtained by the data acquisition system within 1 s, they do not refer to the same point of time. This is because each sensor performs a measurement cycle controlled by its own processor and it cannot be restarted by a software command from the user (or, at least, it is not documented).

This shortcoming is partially worked around by data acquisition hardware, assuming that all AlphaSensor processors have the same oscillator frequency, perform the same measurement cycle and renew output data at a well-defined time moment after power-on. Since the data acquisition system ensures the simultaneous power-on of all sensors, we suppose that the last data from all AlphaSensors correspond to the same unknown earlier moment which lies somewhere between 0 and 10 min before sending the request command (which is already time-synchronized by the server). Nevertheless, a certain time delay remains (see Section 3 below for a more detailed discussion).

Under the assumption that the time profile of AlphaSensor data follows statistically that of AlphaGUARD by a certain time delay (lag), its most probable value corresponds to the maximum of the Pearson correlation coefficient between both series as a function of the time lag between them. This function is well approximated by a parabola in the vicinity of its maximum, whose position is then taken as an estimate for the effective time delay. Finally, each AS data series is translated toward time by its “best lag” value in order to be compared with the AlphaGUARD. Intermediate points in time series are calculated by means of cubic spline interpolation.

#### 2.4.4. Evaluation of the Background

The intrinsic background of the AlphaSensors and that of AlphaGUARD, also, are estimated using data for radon activity measurements in the airtight canister. The decay curve of radon after reaching equilibrium with its daughters is fitted by a simple exponential function:

(1) $$A\left(t\right)=a.e^{-\lambda t}+b$$

where λ = 0.1813 d^−1^ is the decay constant of ^222^Rn. Background is determined by the estimated value of the free parameter b.

#### 2.4.5. Study of Calibration Factor and Linearity

According to the manufacturer, ASs are calibrated for radon-in-air activity concentration to within ±10%. Using AG data as a reference, a plot of AG radon activity concentration values vs. those reported by AS at the same moment t should be a straight line within statistical uncertainties of both measurements:

(2) $$C_{AG}\left(t\right)=k.C_{AS}\left(t\right)+b$$

The intercept b should be statistically zero if the background is evaluated and subtracted from both series. The value of the slope a is used as a calibration factor for each AlphaSensor.

Fitting a data set with uncertainties in both X and Y axes is generally a non-linear least-squares problem, which is considered in detail in [12]. A simplified numerical method [13] was used in this work to approximate activity curve, which is valid, albeit only for straight line fitting models. In this case, if the model function is

(3) y=ax+b

and $\sigma_{x}^{2}$ and $\sigma_{y}^{2}$ are the variances on x and y, then the objective functional to be minimized with respect to the free parameters a and b has the form

(4) $$\chi^{2}\left(a,b\right)=\sum_{i=1}^{n}\frac{(y_{i}{-ax_{i}-b)}^{2}}{a^{2}\sigma_{x_{i}}^{2}+\sigma_{y_{i}}^{2}}$$

Since a also appears in the denominator of Equation (4), then the gradient equation $\frac{\partial \chi^{2}}{\partial a}=0$ is non-linear. However, the corresponding equation for b, $\frac{\partial \chi^{2}}{\partial b}=0$, remains linear. Consequently, the minimization of $\chi^{2}$(a,b) is reduced to a simpler procedure consisting of a one-dimensional non-linear minimization with respect to a, followed by a straightforward calculation of the optimal value of b for each current value of a at every step of the iterative process.

Statistical tests are generally based on the assumption of independent and identically distributed data. However, the time series of radon concentrations reported by the AlphaSensors are significantly correlated, at least due to the internal 60 min smoothing. Therefore, the method of Pyper and Peterman [14] is used to estimate the effective number of degrees of freedom n* for paired data series, each of size *n*:

(5) $$\frac{1}{n^{*}}\approx \frac{1}{n}+\frac{2}{n}\sum_{j=1}^{n}\left(\frac{n-j}{n}\right)\varrho_{XX}(j)\varrho_{YY}(j)$$

where $\varrho_{XX}(j)$ and $\varrho_{YY}(j)$ are the autocorrelation coefficients of the residuals of x and y at lag j. The effective number of degrees of freedom n* was used as a parameter in the chi-squared test of Equation (4) instead of the number of observations *n*.

A measurement value $\left(x_{i},y_{i}\right)$ is considered an outlier if it deviates by more than three standard deviations (σ) from the fit. Outliers are removed (i.e., assigned zero weight) if they occur sporadically and are not successive points within the time series.

## 3. Results and Discussion

### 3.1. Descriptive Statistics

**Figure 3:**
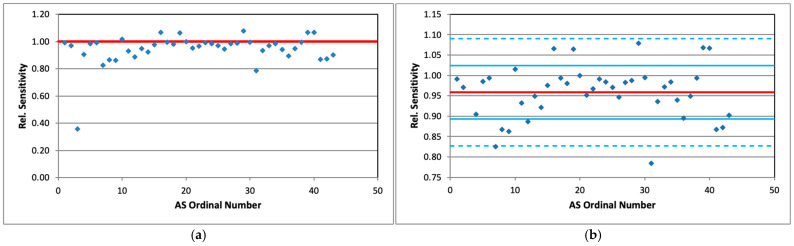
(**a**) Sensitivity of all AS (points) relative to AG (red line); (**b**) the same data with AS03 removed; red line marks the sample mean, blue lines denote ±1σ and ±2σ intervals.

Mean values of the radon concentration activity measured by AlphaSensors during the whole experiment are displayed in Figure 3a relative to those measured by AlphaGUARD. They are a raw estimate for the relative sensitivity of the corresponding AS compared to AG (each AS is given an individual number, AS01 to AS43, for convenience). Statistical uncertainties are much smaller than the point size. It is seen that AS03 value is obviously too low and it was removed from the sample as an outlier. Upon notifying the manufacturer of the AS03 malfunction (reduced sensitivity), the device was promptly replaced at no cost.

The remaining points are shown in Figure 3b together with the ±1σ and ±2σ intervals around the average.

It is seen that one point lies outside 2σ deviation (AS31) and 30 values (71%) are within m ± 1σ, in good accordance with expectations for normally distributed results: Sens = 0.959 ± 0.066.

### 3.2. Assessment of AS Uncertainties

**Figure 4:**
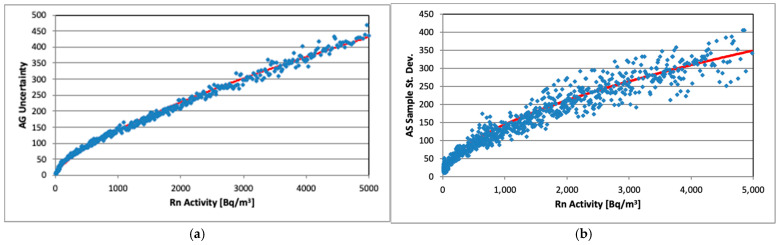
Rn activity uncertainty vs. Rn activity: (**a**) AlphaGUARD (reported uncertainty values); (**b**) AlphaSensor (estimated as sample standard deviation). The red lines show fit results.

As already mentioned, the uncertainty of AG measurements is calculated and reported by the instrument. It is useful to plot the estimated uncertainty of AG and AS as a function of radon activity (Figure 4a,b). The activity range is limited to 0–5000 Bq/m^3^ due to the limited number of measurements available at higher activities (see Figure 1).

Both curves seem rather similar in behavior, although the AG activity concentration uncertainty is the result of an internal calculation by the device, while the AS one is taken as a sample standard deviation including all tested AlphaSensors (except AS03). Several functions were tried to fit the curves imposing two a priori requirements: (a) to be simple, and (b) the same equation to be used for both curves. The best fit was obtained by a power function:

(6) ln(y)=a+b.ln(x)

where a and b are free parameters. The “best” estimates of parameters for AG uncertainty approximation are a = 0.145(6), b = 0.6952(8), and r^2^ = 0.995, and for AS uncertainty, they are a = 1.19(4), b = 0.548(6), and r^2^ = 0.953.

The uncertainty values calculated by Equation (6) are regarded as equally valid for each AS device and are used further to estimate uncertainty of each 10 min measurement. In our opinion, an uncertainty estimate based on a sample standard deviation across all tested sensors at a given moment is more relevant for an end-user since it naturally incorporates both the measurement noise and inter-device reproducibility. Therefore, it is reasonable to recommend its use for AlphaSensors beyond the set examined in this study.

### 3.3. Background Estimation

**Figure 5:**
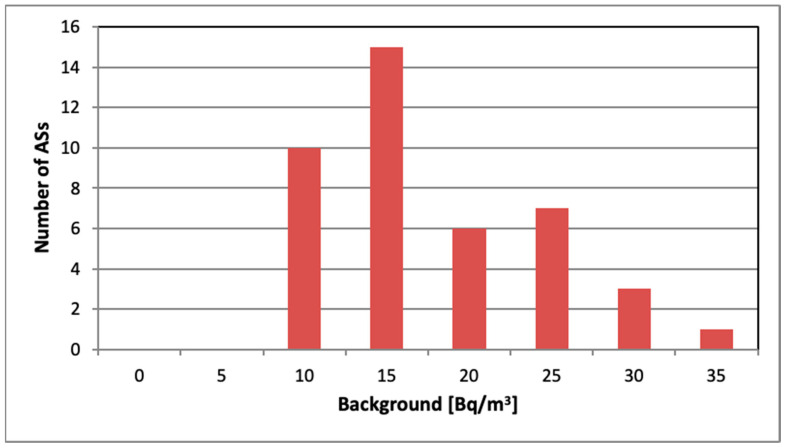
Distribution of background values for ASs.

Data from the 22-day measurement cycle in the airtight vessel were used to estimate the background using the procedure described in Section 2.4.4. The *p*-value for the chi-squared test was greater than 0.05 for all AS radon decay series, except that for AS33, where the measured radon activity did not follow a regular pattern and remained consistently above 100 Bq/m^3^, apparently due to additional electronic noise generated in the device. Figure 5 shows a histogram of all AS radon monitors, excluding AS33 but including AS03, which has lower sensitivity but otherwise functions correctly.

Since the reference radon concentration in the airtight container at the start of measurement was 36(1) Bq/m^3^ and the experiment was conducted in a low radon environment, it is unlikely that the background values obtained are due either to residual radon inside the volume or to a diffusion from outside; they arise partially from decay of long-lived radionuclides or are likely artifacts from spurious counts. The background level assessed for the set of all ASs (except AS33) was equivalent to 15 ± 6 Bq/m^3^. The AG background was 17.6(3) Bq/m^3^.

### 3.4. Time Synchronization of AS Measurements

**Figure 6:**
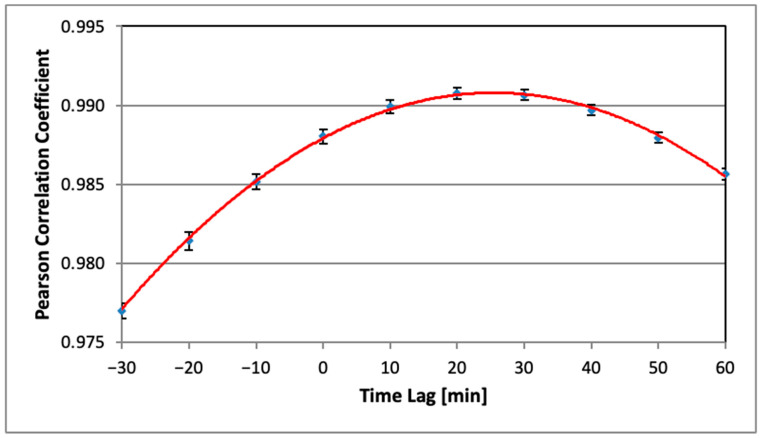
Cross-correlation function between AS01 and AG.

The time synchronization procedure suggested in Section 2.4.3 was implemented for all AS time series. As an example, the essential part of the cross-correlation function between the AG data and one of the AS radon monitors (AS01) is presented in Figure 6.

It has a pronounced maximum and is well fitted by a quadratic parabola (red line in Figure 6). The position of the maximum is taken as the “best” estimate of the time lag (i.e., the most probable value of time delay) between AG and the respective ASxx time series. The latter is then shifted backward in time by the “best lag” to ensure maximum correlation with AG measurements.

**Figure 7:**
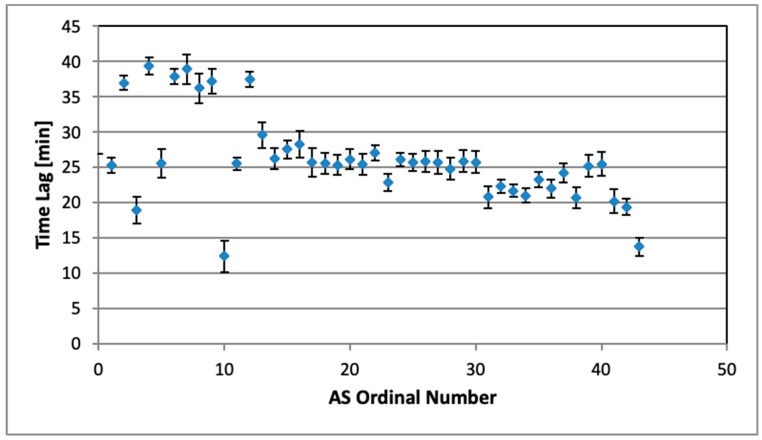
Time lags for all examined AlphaSensors relative to AG.

The values of the best time lags are presented in Figure 7 for all ASs.

Some basic statistics of the time lags and associated correlation coefficients are shown in Table 2.

### 3.5. Linearity and Calibration of ASs

**Figure 8:**
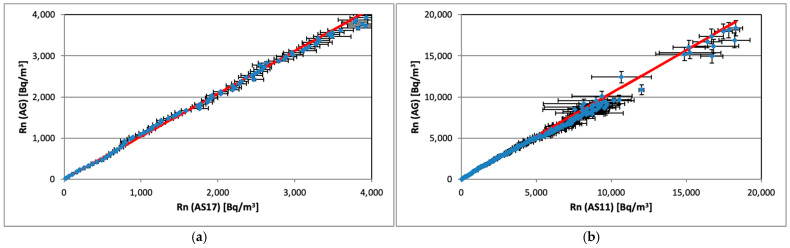
Referent radon concentration activity measured by AG vs. corresponding AS values: (**a**) AS17 (points) and a linear fit (red line) in the range 0–4000 Bq/m^3^; (**b**) AS11 (points) and a linear fit (red line) in the range 0–20,000 Bq/m^3^.

The linearity of each AS was examined by plotting reference AG measurement results against corresponding AS values. The data were fitted with a straight line, taking into account uncertainties in both coordinates. Some examples (Figure 8a,b) illustrate the responses of two ASs and their linear approximation calculated for different activity concentration ranges. All other AlphaSensors show similar results.

**Figure 9:**
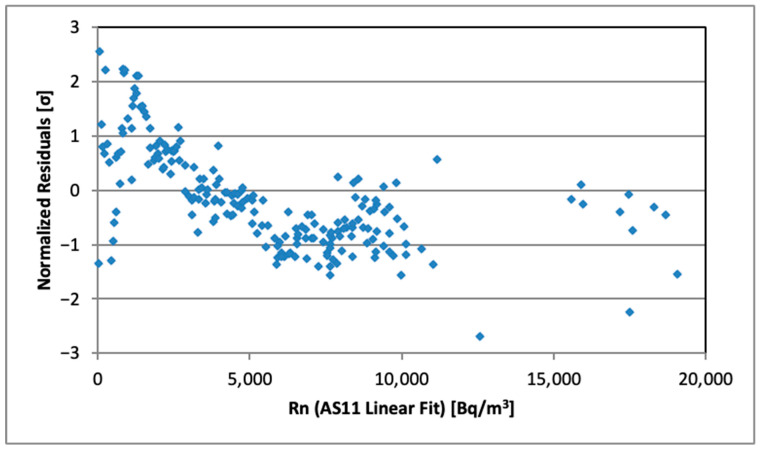
Normalized residuals of AS11 linear fit vs. Rn concentration.

The linear fit was statistically perfect (*p*-value for the chi-squared test was *p* = 1) for all AS in the range 0–4000 Bq/m^3^. The intercepts are approximately zero (sample average is −1.4 ± 2.6 Bq/m^3^) after the estimated background was subtracted from AG and AS measurements. The fit quality parameters remain rather good (*p* = 0.89, normalized chi-squared Χ^2^/*n* = 0.87 for Figure 8b) in the whole studied range up to 20 kBq/m^3^; however, a tiny non-linearity trend of AS values is visible for the values above 4000 Bq/m^3^. This effect is more clearly visible in Figure 9, which presents the plot of residuals (normalized to the estimated standard deviations) for the AS11 data. A similar behavior was observed for all other ASs. Since the linear response of AlphaGUARD is firmly validated, we have no explanation about the origin of this tendency for slightly underestimating the higher activity concentration values by AS (dead time of the detector should be excluded as a possible reason since the ZnS(Ag) scintillation pulses are rather short [15] to assume observable overlapping). In any case its effect (averaged in the range 5–20 kBq/m^3^) was estimated to be 2% of the relative activity concentration.

**Figure 10:**
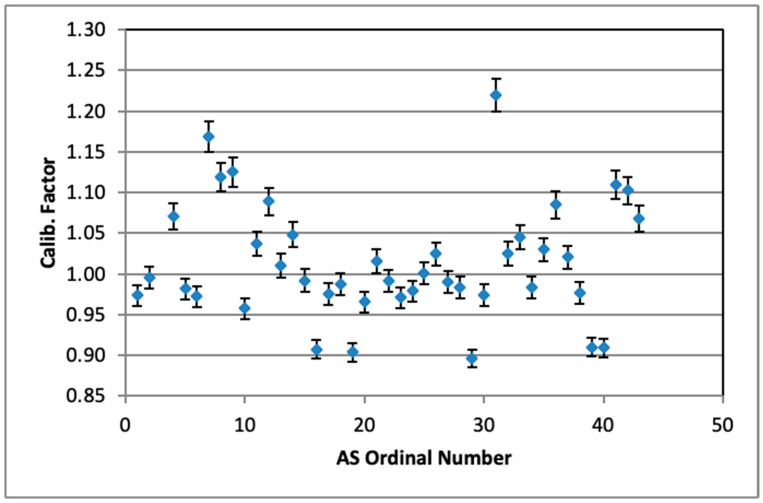
Calibration factors for tested AlphaSensors.

Figure 10 shows the estimated slope of the linear fit, which defines the “best” value of the calibration factor for all AlphaSensors (except AS03). They vary between 0.896 and 1.219, the average value being 1.014 ± 0.071, which is remarkably good.

### 3.6. Time Response of ASs

Characterizing the sensors’ performance through continuous measurements under real environmental conditions facilitates the study of the temporal behavior of the radon activity concentration. A parallel comparison of the time pattern of AS measurements vs. AG ones reveals a marked delay in time and some inertiality of the AlphaSensors’ data.

Besides the already-mentioned unknown time lag between AS and AG measurement cycles, which can be largely compensated by the time synchronization procedure (Section 2.4.3 and Section 3.4), an additional delay between the reactions of both types of instruments to the same impact arises from the diffusion time necessary for radon to enter into the AlphaSensor’s active volume. The AS device operates in passive mode: the atmospheric air, carrying radon gas and radon progeny, diffuses through a narrow, labyrinthine light-tight entrance aperture which stops the progeny but allows the passage of the radon gas. The diffusion time depends on the fine details of sensor construction and may vary slightly for each individual device. On the other hand, AlphaGUARD operates in flow-through mode ensuring virtually prompt air exchange (air flow 1 L/min through 0.56 L volume).

All this would not be important if a stationary stochastic process was observed, but this is not the situation when high dynamics of radon concentration takes place. The weather during the first measurement cycle (in the spring) was rather turbulent and resulted in a large variability of the radon activity concentration at the test site. The rms value of the activity jumps, averaged over the first cycle, is 384 Bq m^−3^ h^−1^, occasionally exceeding 2000 Bq m^−3^ h^−1^ (as a reference, the same value for the third cycle in milder environmental conditions is 82 Bq m^−3^ h^−1^).

**Figure 11:**
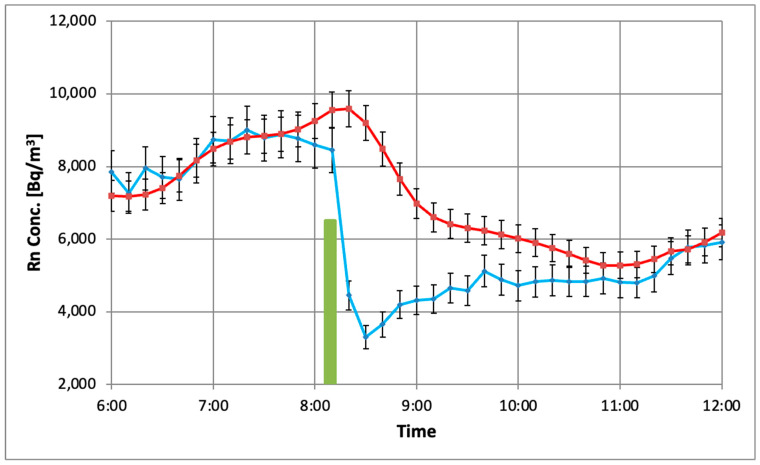
Time response of AG (blue) and AS01 (red) to a short, “spike-wise” activity perturbation (green bar).

In order to study more carefully the time response of the AS, a simple experiment was carried out: the door of the experimental room (1.6 m^2^) was opened wide on March 12th for five minutes between 08:06 and 08:11 GMT. The effect was an immediate drop in the atmospheric pressure inside the room by 30 Pa and a very sharp decrease in the radon concentration (Figure 11).

In contrast to the investigations described above, the data presented in Figure 11 are “as is”, so as to obtain a notion about the effect of a sharp activity change on the end-user. As can be seen, the radon activity reported by the AlphaSensor approaches AG values as late as three hours after the perturbation, in accordance with the time necessary for restoring the equilibrium between ^222^Rn and its progeny in- and outside the AS active volume.

The time delay was estimated by the difference between time positions of the midpoints between the minimum and maximum of the activity curves. The averaged value for all tested AS modules is 35.9 ± 7 min. It has three components: (a) the time lag discussed in Section 2.4.3; (b) the effect of the radon diffusion as well; (c) all that blurred by the 60 min moving average. Taking into account the time lag results listed in Table 2, the additional effect of the diffusion time can be roughly estimated to about 10 min.

Another consequence of the time delay is a distortion of the AS radon concentration activity readout. In the studied case it is equivalent to 5500 Bq·h/m^3^ false higher radon exposure. In a case when radon concentration increases sharply, the AS readout may be false lower than the real value. An estimation based on all measurements in the first cycle indicates relative deviations (ASxx−AG)/AG varying between −30% and +34% even on a daily basis, while the relative deviation averaged for the whole 70-day period is only −2%. This demonstrates once again the advantage of long-term monitoring in obtaining reliable results.

A more precise investigation of the time response is limited by the unknown time lag and by the moving average smoothing of measurement data as well. Some attempts to reveal the time response function from transient data [7] encountered the same problem since it is likewise valid for the RadonEye Plus 2 sensor (Ecosense Inc., San Jose, CA 95125, USA) studied therein. In our opinion, it would be of great benefit for research-grade use of the AlphaSensor module if it is provided with a digital pulse output, which allows direct pulse counting from the user, thus eliminating time delay components (a) and (c) mentioned above.

The following table (Table 3) summarizes the analyses presented in this study and the corresponding AlphaSensor modules that participated in each of them.

## 4. Conclusions

A comparative analysis of 43 AlphaSensor modules against the AlphaGUARD reference monitor demonstrated that AlphaSensor readings differ by approximately 5–10% from those of AlphaGUARD and comply with the values stated by the producer. This deviation can be effectively corrected by applying individual sensitivity coefficients derived for each sensor. The same conclusions are also applicable to all commercial products based on AlphaSensor technology, such as, for example, AlphaTracer radon sensors.

As a general conclusion, the results obtained in the present study confirm that AlphaSensor, due to its affordability and acceptable accuracy after calibration, is suitable for deployment in large-scale monitoring networks. Such networks can serve both indoor radon surveillance for health risk mitigation and radon in soil gas monitoring as a geophysical tracer associated with seismic processes. A design-related drawback of the sensor is its relatively low sensitivity, which limits its applicability in portable instruments intended for rapid radon measurements—a capability achievable only with substantially more expensive devices. The lack of time synchronization and the reporting of only internally smoothed radon concentration values also constrain its performance in research applications. These limitations, however, could be mitigated through modest hardware refinement.

## Figures and Tables

**Table 1:** Main technical characteristics of AlphaSensor and AlphaGUARD.

Device	Operation Mode	Range [Bq/m^3^]	Sensitivity [cpm/kBq.m^−3^]	Accuracy [%]	Precision [%]
AlphaSensor module	Passive (diffusion)	2–2,000,000	2.83	10	<±10@370 Bq/m^3^ after 1 h
AlphaGUARD 1000PF	Active (AlphaPUMP)	1–1,000,000	50	3	<±10@200 Bq/m^3^

**Table 2:** Sample statistics of best values for time lags between AG and AS.

Statistic	Best Lag Value [Min]	Pearson (r)
Average	26.1	0.983
St. Dev.	6.1	0.007
Min	12.5	0.969
Max	39.4	0.993

**Table 3:** Summary of the analyses performed with the AlphaSensors.

Section	Used AS	Excluded AS	Remark
Section 3.1	42	1 (AS03)	Low sensitivity
Section 3.2	42	1 (AS03)	Low sensitivity
Section 3.3	42	1 (AS33)	Non-radiative noise
Section 3.4	42	1 (AS03)	AS03 Low sensitivity AS33 Linear above 300 Bq/m^3^
Section 3.5	42	1 (AS03)	Low sensitivity
Section 3.6	42	1 (AS03)	Low sensitivity

## Data Availability

The complete raw data set can be downloaded from https://doi.org/10.5281/zenodo.18420947.

## Abbreviations

The following abbreviations are used in this manuscript:

AG	AlphaGUARD
AS	AlphaSensor/s
Rms	Root mean square
Rn	Radon (chemical element)
